# Mitochondrial Dysfunction and Oxidative Stress in Liver Transplantation and Underlying Diseases: New Insights and Therapeutics

**DOI:** 10.1097/TP.0000000000003691

**Published:** 2021-10-21

**Authors:** Shaojun Shi, Ling Wang, Luc J.W. van der Laan, Qiuwei Pan, Monique M. A. Verstegen

**Affiliations:** 1 Department of Surgery, Erasmus MC—University Medical Center, Rotterdam, the Netherlands.; 2 Department of Gastroenterology and Hepatology, Erasmus MC—University Medical Center, Rotterdam, the Netherlands.

## Abstract

Mitochondria are essential organelles for cellular energy and metabolism. Like with any organ, the liver highly depends on the function of these cellular powerhouses. Hepatotoxic insults often lead to an impairment of mitochondrial activity and an increase in oxidative stress, thereby compromising the metabolic and synthetic functions. Mitochondria play a critical role in ATP synthesis and the production or scavenging of free radicals. Mitochondria orchestrate many cellular signaling pathways involved in the regulation of cell death, metabolism, cell division, and progenitor cell differentiation. Mitochondrial dysfunction and oxidative stress are closely associated with ischemia-reperfusion injury during organ transplantation and with different liver diseases, including cholestasis, steatosis, viral hepatitis, and drug-induced liver injury. To develop novel mitochondria-targeting therapies or interventions, a better understanding of mitochondrial dysfunction and oxidative stress in hepatic pathogenesis is very much needed. Therapies targeting mitochondria impairment and oxidative imbalance in liver diseases have been extensively studied in preclinical and clinical research. In this review, we provide an overview of how oxidative stress and mitochondrial dysfunction affect liver diseases and liver transplantation. Furthermore, we summarize recent developments of antioxidant and mitochondria-targeted interventions.

## INTRODUCTION

The liver plays a pivotal role in detoxification and blood purification to protect the organism from endogenous and exogenous toxic compounds. Related to its high metabolic activity, liver parenchymal/epithelial cells including hepatocytes and cholangiocytes contain high numbers of mitochondria. These mitochondria maintain homeostasis of cellular energy but also contribute hepatic pathogenesis once their metabolic function is impaired. Components of electron transport chain (ECT) of mitochondrial can be affected by numerous triggers such as drugs, high lipid intake, viruses, or ischemia.^1^ Mitochondrial perturbation results in oxidative stress, defined as imbalanced redox homeostasis caused by excessive synthesis of oxidative products or exhaustion of antioxidant substances. Oxidative substances, mostly reactive oxygen species (ROS) and reactive nitrogen species (RNS), are mainly produced in parenchymal cells and Kupffer cells in the liver and can damage biologically relevant macromolecules such as carbohydrates, DNA, proteins, and lipids.^2^ Mitochondrial dysfunction and oxidative stress are inseparable processes and play a critical role in cell death, inflammation and fibrosis during many liver diseases such as steatohepatitis, alcoholic liver disease (ALD), cholestasis, cirrhosis, and hepatic malignancies.^3^ Also, ischemia-reperfusion injury (IRI), a major cause of liver transplantation failure, is also strongly associated with oxidative injury.^4^ However, the crosstalk between redox signaling and mitochondrial defects and its precise role in hepatic pathogenesis remain unclear. In addition, over the last decades, various therapies have been developed to eliminate ROS/RNS, enhance antioxidant defense or restore mitochondrial homeostasis. Several antioxidative agents, such as vitamin E and ursodeoxycholate (UDCA), have been clinically utilized as the first-line drugs in the treatment of liver diseases.^5^ Nevertheless, numerous antioxidative therapies failed to get into clinical application due to unsatisfactory clinical outcome or even side effects in patients. This phenomena, also known as the “antioxidant paradox,” highlights the gaps in our current knowledge and the incomplete understanding of the pathogenetic role of mitochondrial dysfunction and oxidative stress in liver transplantation and underlying diseases.^6^ In this review, we first summarize the evolving concepts and underlying mechanisms of mitochondrial impairment and redox imbalance and interpret their involvement in cell death, inflammation, and fibrogenesis. We then emphasize the pathogenetic role of mitochondrial dysfunction and oxidative stress in major liver diseases and liver transplantation. Finally, the recent advancement of antioxidative and mitochondria-targeted therapeutic regimens will also be discussed.

## MITOCHONDRIAL DYSFUNCTION AND OXIDATIVE STRESS IN HEPATO-PATHOGENESIS

### Mitochondria as the Main Source of ROS/RNS

The ROS/RNS family includes superoxide (O_2_^−^), hydrogen peroxide (H_2_O_2_), peroxynitrite, and nitric oxide (NO).^7^ Continuous generation of ROS/RNS is a part of normal aerobic metabolism and serves as signal-transducing molecules in metabolism, gene transcription/translation, cell cycle, and differentiation.^5^ Excessive ROS/RNS are scavenged by the antioxidant defense system. The imbalance between ROS/RNS production and scavenging, however, is detrimental to cells. The shift from being beneficial to detrimental is orchestrated by the amount and duration of ROS/RNS production.^8^

**Figure 1.**
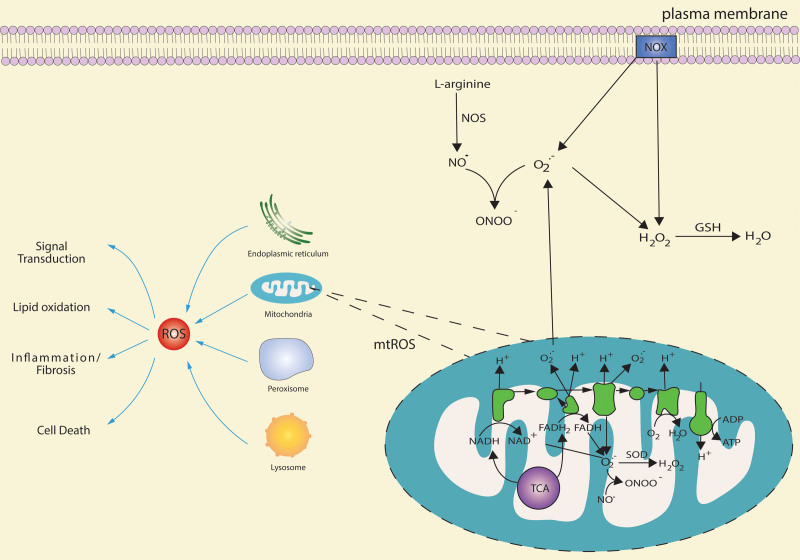
The main intracellular sources of ROS/RNS. Many cell organelles can produce ROS, acting as a signal-transducing molecule. Excessive ROS can induce lipid oxidation, inflammatory responses, fibrosis, or cell death in the liver. The main source of ROS is generated by ETC, NADH, and FADH^2^ are respectively involved in the TCA cycle, and β-oxidation of fatty acids donates electron and H^+^ to mitochondrial ETC. With the process of electron transfer from complex I/complex II to complex IV, electrons leak from ETC attack O_2_, leading to O_2_^−^ formation. O_2_^−^ has a short half-life that is rapidly divided into H_2_O_2_ and O_2_ by SOD. NOX is also the main ROS source that transfers an electron to O_2_ to produce O_2_^−^ and H_2_O_2_. Increasing mtROS activate NOXs, which, in turn, enhances mtROS generation. This feed-forward cycle between NOXs and mitochondria maintains the cellular redox homeostasis. O_2_^−^ produced by ETC can respond to NO^•^, which is produced from L-arginine by NOS catalysis to generate ONOO^−^. Besides, ONOO^−^ can also be produced in mitochondria, which is mainly catalyzed by mtNOS. ETC, electron transport chain; FADH2, flavin adenine dinucleotide; mtNOS, mitochondrial NOS; ONOO^−^, peroxynitrite; NADH, nicotinamide adenine dinucleotide; NADPH, nicotinamide adenine dinucleotide phosphate; NOXs, NADPH oxidases; RNS, reactive nitrogen species; ROS, reactive oxygen species; SOD, superoxide dismutase; TCA, tricarboxylic acid.

Mitochondria are intracellular organelles serving as cellular powerhouses and are the main source of ATP production in a cell. In addition to this role in energy metabolism, mitochondria are involved in calcium homeostasis, signal transduction, and controlling programmed cell death.^9^ Most intracellular ROS/RNS are produced by impaired mitochondria^10^ as byproducts of oxidative metabolism (Figure 1). They are mainly generated at the site of oxidative phosphorylation (OXPHOS) of the ETC, which takes place on the inner mitochondrial membrane.^11,12^ Approximately 0.2%–2.0% of electrons still escape from complex I and III of ETC.^13^ Electron leakage generates O_2_, which is rapidly divided into H_2_O_2_ and O_2_ by superoxide dismutase (SOD).^12^

Another primary source of ROS is through nicotinamide adenine dinucleotide phosphate oxidases (NOXs), which catalyzes electron transfer from nicotinamide adenine dinucleotide phosphate to molecular oxygen to produce O_2_^−^ and H_2_O_2_.^14^ Activated NOXs increase mitochondrial ROS (mtROS) production, and increased mtROS level, in turn, activates NOXs, suggesting a feed-forward cycle between mtROS and NOXs.^15,16^

The RNS family, including NO, also plays a critical role in liver redox reactions. NO is produced from L-arginine catalyzed by NO synthases. Mitochondrial NO synthases activity is regulated by complex I of ETC,^17,18^ and mitochondria have been suggested as a primary source of RNS.^19^

### Mitochondrial Dysfunction is Associated With Programmed Cell Death

**Figure 2.**
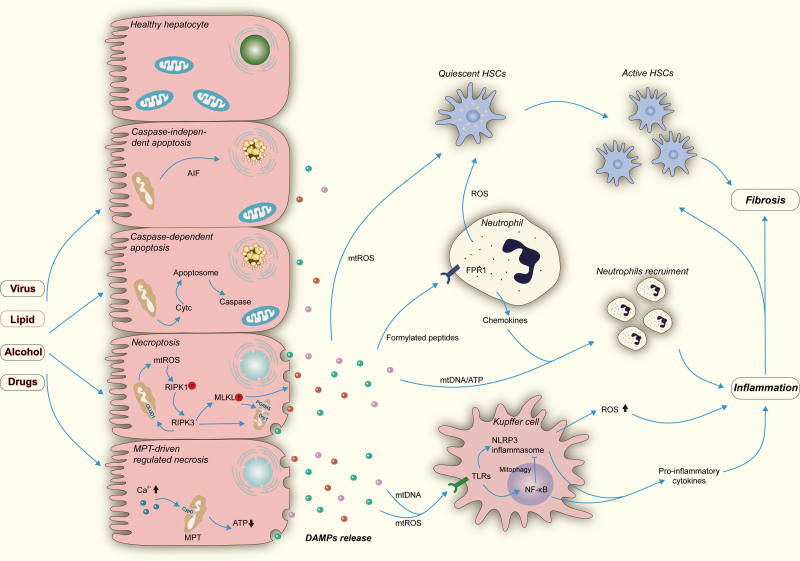
The role of oxidative stress and mitochondrial dysfunction in hepatic cell death, inflammation, and fibrogenesis. A, Mitochondrial function could be impaired during chronic liver injury induced by the hepatitis virus, fatty acid, alcohol, and drugs. Defected mitochondrial and subsequent oxidative stress are involved in the execution of multiple programmed cell death. Proapoptotic proteins such as AIF could translocate from mitochondrial to the cytosol via permeabilization of the mitochondrial membrane and ultimately elicit caspase-independent apoptosis. Likewise, released Cytc from mitochondrial can induce caspase-dependent apoptosis by forming apoptosome and activating caspase effectors. mtROS released from impaired mitochondrial facilitates the auto-phosphorylation of RIPK1 and thus promotes necroptosis. RIPK3 could result in a massive generation of ROS by activating mitochondrial GLUD1. MLKL could mediate fragmentation of mitochondrial through activation of PGAM5 and Drp1 and consequently execute necroptosis. Besides, overloaded Ca^2+^ triggers CYPD-mediated MPT, leading to a dramatic drop of ATP and ultimately causes MPT-driven regulated necrosis. Subsequent released DAMPs from dying cells are critical effectors in hepatic inflammation and fibrogenesis. B, mtDNA and mtROS can interact with TLRs on Kupffer cells, activating NF-κB pathways and NLRP3 inflammasomes, promoting the production of pro-inflammatory cytokines. Activated NF-κB could, in turn, suppress NLRP3 inflammasomes by the elimination of defective mitochondrial, mediated by mitophagy. Activated Kupffer cells serve as an essential ROS source, amplifying inflammation and spreading cell death. C, Mitochondrial formylated peptides conjunct with FPR1 on neutrophils, which promotes neutrophil chemotaxis. Chemokines collaborated with formylated peptides, ATP and mtDNA, lead to neutrophils recruitment. D, ROS produced by Kupffer cells, neutrophils, and injured hepatocytes could transform HSCs from quiescent to functional status and further promote the proliferation of active HSCs, consequently causing hepatic fibrosis. AIF, apoptosis-inducing factor; CYPD, cyclophilin D; Cytc, cytochrome c; DAMPs, damage-associated molecular patterns; Drp1, dynamin-related protein 1; FPR1, formyl peptide receptor 1; GLUD1, glutamate dehydrogenase 1; HSC, hepatic stellate cell; MLKL, pseudokinase mixed lineage kinase domain-like; MPT, mitochondrial permeability transition; mtDNA, mitochondrial DNA; mtROS, mitochondrial ROS; NF-κB, nuclear factor kappa B; NLRP3, NOD-, LRR- and pyrin domain-containing protein 3; PGAM5, phosphoglycerate mutase family member 5; RIPK1, receptor-interacting protein kinase 1; RIPK3, receptor-interacting protein kinase 3; ROS, reactive oxygen species; TLRs, toll-like receptors.

Cell death is a crucial feature of liver injury. Mitochondrial dysfunction and oxidative stress contribute to apoptosis, a well-characterized type of programmed cell death. Increased mtROS/RNS oxidize cardiolipin, a mitochondrial lipid, which augments mitochondrial membrane permeability and the release of cytochrome c (Cytc). Released Cytc induces the formation of apoptosome complexes and subsequent activation of caspase-3 and -9, leading to caspase-dependent apoptosis (Figure 2).^20,21^ Alternatively, pro-apoptotic proteins, such as apoptosis-inducing factor (AIF), translocated from the mitochondria to the cytosol through permeabilization of the mitochondrial membrane, mediating the fragmentation of DNA and causing caspase-independent apoptosis (Figure 2).^22^

There are several nonapoptotic forms of programmed cell death, also known as “regulated necrosis,” including necroptosis, ferroptosis, pyroptosis, and mitochondrial permeability transition-driven necrosis. These nonapoptotic programmed cell death share similar morphologic features with accidental necrosis but are regulated by specific signaling.^23^ Emerging evidence suggests a critical role of nonapoptotic programmed cell death in hepatic pathogenesis.^23,24^ Mitochondrial dysfunction essentially contributes to MPT-driven programmed cell death (Figure 2),^25^ but not ferroptosis, an iron-dependent form of programmed cell death.^26,27^

The role of mitochondrial dysfunction and oxidative stress in programmed cell death is multifaceted. For instance, necroptosis is mediated by receptor-interacting protein kinase 1, receptor-interacting protein kinase 3, and pseudokinase mixed lineage kinase domain-like, in the case caspase 8 activity is absent or blocked. MtROS functions in a positive feedback loop to promote necroptosis by facilitating the auto-phosphorylation of receptor-interacting protein kinase 1 and necrosome formation. A burst of ROS could be triggered by receptor-interacting protein kinase 3 via the activation of mitochondrial glutamate dehydrogenase 1 and serves as an effector of necroptosis.^28,29^ Pseudokinase mixed lineage kinase domain-like activity may result in mitochondria fragmentation by activating phosphoglycerate mutase family member 5 and dynamin-related protein 1 to execute necroptosis (Figure 2).^30^ Nonetheless, recent studies suggest that necroptosis cannot be retarded by the depletion of either mitochondria or phosphoglycerate mutase family member 5, implicating that mitochondria do not play a role in necroptosis.^31,32^ MtROS production is supposed to accompany, rather than cause, necroptosis.^32^ Hence, there may be multiple mechanisms involved in necroptosis, and the precise role of mitochondrial dysfunction and oxidative stress remains to be further studied.

### Mitochondrial Dysfunction and Oxidative Stress in Hepatic Inflammatory Response

Mitochondrial dysfunction and oxidative stress participate in hepatic inflammatory responses via damage-associated molecular patterns (DAMPs) released from damaged hepatocytes. DAMPs bind to various cell surface or intracellular receptors to activate innate and adaptive immune responses.^33^ In parallel, passive release or active secretion of mitochondria DAMPs (mtDAMPs) into cytosol or extracellular space trigger inflammatory responses by directly binding to the cell surface and intracellular receptors.^34^ Interaction of mitochondrial DNA (mtDNA) and mtROS with toll-like receptors results in activation of pro-inflammatory signaling through nuclear factor kappa B, NOD-, LRR- and pyrin domain-containing protein 3 inflammasomes.^35,36^ Passively released mitochondrial formylated peptides from necrotic cells can interact with formyl peptide receptor 1 on neutrophils, driving neutrophil chemotaxis and neutrophil-dominant sterile inflammation.^34,37^ Mitochondrial formyl peptides, ATP, and mtDNA can collaborate with chemokines including chemokine (C-X-C motif) ligand-1 (CXCL1) and -8 (CXCL8) to mediate neutrophil recruitment. This further amplifies hepatocyte necrosis and may elicit a systemic inflammatory response and remote tissue injury.^38^ mtROS also serves as a secondary messenger to regulate inflammasome-(in)dependent pro-inflammatory cytokine production by modulating the equilibrium between positive and negative regulators.^39,40^ NF-κB has been shown to restrain NOD-, LRR- and pyrin domain-containing protein 3 activation through mitophagy by deposition of dysfunctional mitochondria, revealing a critical anti-inflammatory mechanism of mitophagy (Figure 2).^41,42^

### Mitochondrial Dysfunction in Liver Regeneration and Fibrosis

The liver has remarkable regenerative capacity to maintain tissue homeostasis when exposed to toxins or partial hepatectomy.^43^ Currently, little is known about the role of mitochondrial dysfunction and oxidative stress in liver regeneration, which is controlled by the delicate balance of hepatocyte division and apoptosis. Typical compensatory cellular hyperplasia is accompanied by increased mitochondrial oxidation. However, a recent study revealed impaired mitochondrial function upon the arrest of hepatocyte division. To maintain liver size, synthesis of alanine and a-ketoglutarate from pyruvate is boosted to induce compensatory cellular hypertrophy, rather than hyperplasia.^44^

In contrast to a “transient” regenerative response evoked by acute cellular injury, chronic injury by alcohol, fatty acid, or virus promotes a fibrogenic response characterized by inflammation and remodeling of the liver extracellular matrix. Mitochondrial dysfunction and oxidative stress have been recognized as critical mediators of liver fibrosis.^45^ ROS produced by hepatocytes and Kupffer cells as a response to damage is involved in cell death and subsequent amplification of paracrine inflammation to promote fibrogenesis.^46^ Hepatic stellate cells (HSCs) are the main effectors in liver fibrosis through the transformation from quiescent to a functional state in response to damage. HSC activation involves the excessive generation of ROS, mainly NOX-dependent ROS, in neighboring cells such as neutrophils, Kupffer cells, and damaged hepatocytes.^47^ However, once HSCs are activated, their response to ROS is deflected. ROS provides a critical proapoptotic trigger to activated HSCs, possibly by decreasing intracellular glutathione (GSH) levels.^48-50^ As current antifibrotic therapies are based on using antioxidants, this paradox should be critically assessed. Of note, a recent study demonstrates that mtDAMPs released from injured hepatocytes are regulated by phagocytic hepatic macrophages and could directly activate HSCs, thus promoting liver fibrosis.^51^ This suggests that direct targeting mtDAMPs or modulating mtDAMPs by phagocytic hepatic macrophages may be a potential antifibrotic strategy.

## MITOCHONDRIAL DYSFUNCTION AND OXIDATIVE STRESS IN LIVER TRANSPLANTATION

Liver transplantation using conventional organ procurement and preservation methods is always associated with IRI. In addition to liver transplantation, hepatic IRI is a major risk in clinical settings such as shock, trauma, and liver resection. The damage to parenchymal cells is elicited by deprivation of oxygen and becomes augmented once the oxygen supply is restored. In particular, hepatic warm and cold ischemia followed by reperfusion in liver transplantation represents the main factor determining graft function and survival after transplantation.^52^ The mechanism of hepatic IRI encompasses exacerbated oxidative injury, impairment of mitochondrial function, and activation of the immune system. Clinical evidence has shown that circulating mtDAMPs are associated with the onset of early allograft dysfunction in liver transplant recipients.^53^ In light of organ shortage, grafts from extended criteria donors, such as older, steatotic, and brain death donors, have been increasingly applied in liver transplantation. These are considered to be more susceptible to IRI, especially as a result of oxidative injury.^54^

Interruption of the mitochondrial respiratory chain in hepatocytes lacking sufficient oxygen is the first event in (warm) ischemia. Consequently, OXPHOS is abrupted quickly, which causes depletion of ATP, acceleration of glycolysis, and production of lactate, leading to hepatocyte death and DAMPs release.^55^ Sinusoidal endothelial cells play a protective role in liver homeostasis and remain the most injured cell type in cold ischemia, also known as “preservation injury,” which can induce ROS production and further Kupffer cell activation.^55^ Recent studies show that endothelial dysfunction during cold ischemia is mediated by downregulation of Kruppel-like Factor-2, a cellular growth and differentiation regulator, which impairs the antioxidant system by suppressing transcription of protective genes such as endothelial synthase of NO and Nrf2.^56,57^ Multiple studies revealed the beneficial effect of pretreatment of donors after circulatory death with Kruppel-like Factor-2 inducers as a supplement in the cold storage solution, attenuating oxidative stress and improving graft liver function.^57,58^ Subsequently, reperfusion injury is initiated by ischemia and represents a dramatically vulnerable phase with massive oxidative injury and inflammatory response. A major event in this phase is the excessive production of ROS in the recovered and viable cells.^59^ In the early stage of reperfusion, damaged endothelial cells and Kupffer cells are the key cell type producing ROS, which further actives Kupffer cells and initiates neutrophil recruitment, ultimately leading to a larger portion of damage and inflammatory response in the late stage of reperfusion.^60^ Emerging evidence indicates that the burst of ROS generation and oxidative stress in grafts from extended criteria donors under IRI stress could be ameliorated by ex vivo machine perfusion.^61^ Moreover, both antioxidative treatment of donor and recipient and supplementation of antioxidants during machine perfusion exhibit promising therapeutic potential to diminish hepatic IRI.^62,63^

Defective mitochondria play a critical role in hepatic IRI. Mitochondrial permeability transition pore (MPTP) opening could be induced by alkaline pH, mtROS (rather than cytosolic ROS), and calcium overload during IRI, leading to depolarization, uncoupling and swelling of mitochondria and ultimately cause ATP depletion and necrosis, which is the dominant cell death type in reperfusion phase.^4^ Primarily, after the onset of MPTP, the subsequent extracellular release of Cytc can trigger ATP- and caspase-dependent apoptosis. However, once ATP is depleted due to sudden ischemia, necrotic cell death is induced in hepatocyte. Thus, in the context of hepatic IRI, the depletion status of ATP serves as a switch between ATP-dependent apoptosis and ATP depletion-dependent necrosis.^64^ As mentioned previously, mtDAMPs released from dead cells are capable to trigger localized and systematic inflammation. A recent study suggests that the post-transplant level of serum mtDNA fragment is associated with the recovery of patients undergoing liver transplantation and predicts the onset of postoperative multiorgan dysfunction syndrome.^65^ Additionally, blockage of MPTP to restore mitochondrial function has been studied for a long time as a promising therapeutic approach against hepatic IRI. Cyclophilin D is a critical regulator of MPTP opening in the inner mitochondrial membrane. A recent study demonstrated that the small-molecule inhibitors of cyclophilin D reduce calcium-induced mitochondria swelling, restore hepatic calcium retention and OXPHOS parameters, and consequently attenuate hepatic IRI.^66^

Innate and adaptive immune responses are strongly associated with immediate graft function and long-term outcome in liver transplantation. The involvement of mitochondrial in this process cannot be ignored. The metabolism and functions of innate and adaptive immune cells are extensively modulated by mitochondria, in which mtROS acts as the critical signaling molecules. For instance, excessive ROS and perturbation of mitochondrial function are detrimental to neutrophil migration and promote neutrophils apoptosis.^67,68^ Similar results are seen in macrophages, where defective mitochondria prevent repolarization from a pro-inflammatory macrophage phenotype to an anti-inflammatory phenotype.^69^ In contrast, the production of mtROS is not detrimental, but an essential step for T cells activation.^70^ Likewise, mitochondrial function instructs the activation and cell fates of B cells.^71^ A recent study observed an increased expression of mitochondrial-encoded genes in peripheral blood mononuclear cell of recipients with acute rejection after kidney transplantation, potentially indicating a predictive value of mitochondrial gene expression for the onset of rejection.^72^ In rodent orthotopic liver transplant model, oxidative DNA damage and mtDNA mutation in hepatocytes were found to be caused by tumor necrosis factor-alpha, which further promotes graft rejection.^73^

## MITOCHONDRIAL DYSFUNCTION AND OXIDATIVE STRESS IN LIVER DISEASES

### Nonalcoholic Fatty Liver Disease

The burden of nonalcoholic fatty liver disease (NAFLD) is rapidly growing worldwide.^74^ Alterations of mitochondrial morphology and function have been widely observed in hepatocytes from NAFLD patients and animal models.^75^ At the early stage of NAFLD, increasing lipid levels are accompanied by higher numbers of mitochondria. This is associated with increased OXPHOS, hepatic cardiolipin, ubiquinone, and mitochondrial DNA.^76-79^

Mitochondrial dysfunction and oxidative stress feature the progression from steatosis to nonalcoholic steatohepatitis (NASH). In these patients, mitochondrial adaptation is impaired possibly because of increased ROS levels and the triggered inflammatory response.^80^ In NASH patients, hepatic mitochondria have a swollen, rounded shape, and multilamellar membranes with loss of cristae and accumulation of paracrystalline inclusion bodies.^75,81^ These morphologic changes might involve membrane permeabilization and mitochondrial impairment. Hepatic cardiolipin, a unique phospholipid located at the inner membrane interacts with many other mitochondrial inner membrane proteins, enzymes, and metabolite carriers, and is particularly susceptible to ROS-induced oxidation.^82-84^ Oxidative cardiolipin affects ETC complex activity, ETC super-complex stability, and cooperates with Ca^2+^ to induce MPTP opening.^85-87^ Moreover, cardiolipin provides a recognition site for Bcl2 proteins to mediate apoptosis.^88^

Uncoupling protein plays a vital role in ETC uncoupling to regulate ATP synthesis, the NAD+/NADH ratio, and other metabolic pathways.^89^ Uncoupling protein-2 (UCP2) is a mitochondrial inner membrane protein related to oxidative stress.^90^ Under physiologic conditions, UCP2 is expressed in Kupffer cells rather than hepatocytes.^91^ However, it becomes abundant in hepatocytes of fatty liver.^92^ Suppression of UCP2 limits hepatic IRI in ob/ob mice.^93^ These findings indicate that UCP2-dependent mitochondria uncoupling seems an essential factor in NASH development.

### Alcoholic Liver Disease

Continuous consumption of alcohol drives hepatocyte-injury and liver inflammation. ALD can progress to fibrosis, cirrhosis, and the development of hepatocellular carcinoma (HCC).^94^ Similar to NAFLD and NASH, ALD involves mitochondrial dysfunction and oxidative stress.

There are 2 major oxidative pathways of alcohol metabolism in the liver. Ethanol is converted to acetaldehyde by alcohol dehydrogenase, or is metabolized by cytochrome P-450 2E1 through the microsomal ethanol-oxidizing system.^3,95^ Hepatic mitochondria are vulnerable targets by ethanol, which consumes NAD+ leading to increased NADH/NAD+ ratio. This inhibits the TCA cycle and fatty acid oxidation in the liver and stimulates lipogenesis to cause steatosis.^3,96^ Alternatively, acetaldehyde, the metabolite of ethanol, induces mitochondrial damage and cytotoxicity through increasing ROS and Ca^2+^ levels.^97^ Oxidative stress, triggered by acetaldehyde-induced mitochondrial functionality loss, sensitizes hepatocytes to further oxidative damage.^98^

As a result of chronic alcohol intake, lipopolysaccharide is released from the gut^99,100^ and increases ROS production. This induces mtDNA oxidative damage and inhibits mitochondrial gene transcription, eventually leading to mitochondrial dysfunction.^101^ In parallel, lipopolysaccharide also activates Kupffer cells that produce large amounts of ROS, chemokines, and pro-inflammatory cytokines augmenting inflammation, fibrosis, and cell death.^99,102^

### Viral Hepatitis

Hepatitis B virus (HBV) and hepatitis C virus (HCV) infections are frequently accompanied by mitochondrial injury.^103,104^ These infections promote mitochondrial fission by inducing dynamin-related protein 1 phosphorylation and induce Parkin-dependent mitophagy.^103,105^ But they have different ways to alter mitochondrial functions. HBV infection induces alterations of mitochondrial functions and transmembrane potential, increases mtROS levels, and regulates calcium homeostasis.^106-108^ HCV infection stimulates fatty acid synthesis to support viral RNA replication^109,110^ and overload of fatty acids triggers mitochondrial damage. NS5A, an HCV nonstructural protein, activates NF-κB and STAT-3 by inducing oxidative stress and alteration of calcium levels,^111^ resulting in inflammatory response and subsequent HCC induction.^112^

### Drug-induced Liver Injury

Acetaminophen overdose is the most frequent cause of drug-induced liver failure in Western medicine.^113^ Mitochondrial dysfunction and oxidative stress play a critical role in acetaminophen hepatoxicity.^114^ Generally, acetaminophen is metabolized into the reactive metabolite, N-acetyl-p-benzoquinone imine, primarily by cytochrome P-450 2E1, which can be deposed by GSH under a therapeutic dose of acetaminophen. In the context of acetaminophen overdose, N-acetyl-p-benzoquinone imine is continuously generated due to the overwhelmed GSH, which contributes to the depletion of GSH, the formation of adducts and subsequently binds to mitochondrial proteins, causing mitochondrial dysfunction and increased ROS/RNS generation.^3^ N-acetylcysteine, the GSH precursor, has been introduced and clinically applied for the treatment of acetaminophen hepatoxicity since the 1970s.^115^ It replenishes GSH stores, improves hemodynamics, scavenges ROS/RNS, recovers mitochondrial GSH, and supports mitochondrial bioenergetics.^116,117^ Moreover, excessive ROS/RNS actives mitogen-activated protein kinases, especially c-jun N-terminal kinase (JNK) whereby phosphorylated JNK interplays with mitochondrial and augments oxidative stress and further promotes MPTP opening and release of AIF.^118^ Oncolytic necrosis, a dominant type of cell death in acetaminophen hepatotoxicity, can be induced by AIF and elicits the release of DAMPs, including mtDAMPs, inducing sterile inflammation.^34^ Circulating glutamate dehydrogenase, mtDNA and nDNA fragments are useful indicators to evaluate mitochondrial damage in acetaminophen hepatoxicity and are strongly associated with the outcome in patients.^34^

### Cholestatic Liver Diseases

Cholestatic liver diseases (CLD) are featured by an overload of detrimental bile acid in liver and circulation due to alteration of bile formation or intrahepatic/extrahepatic obstruction of bile ducts.^119^ Several studies uncovered the central role of mitochondrial dysfunction and oxidative stress in CLD. A striking increase of oxidative injury markers has been reported in patients with chronic CLD, while the levels of antioxidants decreased.^120^ Similar results were found in bile duct ligated rats, an experimental CLD model, in which a systemic elevation of lipid peroxidation products occurred in the liver and extrahepatic tissues such as kidney and heart.^121^ In the context of moderate CLD, intrahepatic accumulation of low concentrations of hydrophobic bile acid triggers hepatocyte apoptosis via massive production of mtROS.^122^ With regard to severe CLD, dramatically increased levels of bile acid triggers necrosis mainly by lipid peroxidation.^123,124^ Additionally, MPT can be induced and thus impairs mitochondrial OXPHOS, initiating disruption of mitochondria.^123,125^ It has been shown that inhibition of mitochondrial fission effectively diminishes ROS levels, hepatocellular injury, and fibrosis in CLD.^126^ Nuclear factor-erythroid 2-related factor-2 (Nrf2), a critical regulator of antioxidative genes, has been identified as a potential target for treating CLD. Sustained activation of Nrf2 shows hepatoprotective effects in experimental CLD models.^127^ One of the critical beneficial mechanisms of UDCA is believed to stimulate Nrf2, which subsequently restores detoxification and antioxidative defense systems in the liver.^128^

### Liver Cancer

Primary liver cancer, mainly including HCC and cholangiocarcinoma (CCA), becomes the second major cause for cancer-related mortality worldwide.^129^ HCC and CCA are highly heterogeneous and featured by aggressive progression and poor prognosis, but the underlying mechanisms remain elusive. Accumulating evidence suggests that mitochondrial dysfunction and oxidative stress act as initiator or promoter of the tumor initiation, progression, recurrence, and metastasis.^130^ Toxic mtROS is produced in aberrant mitochondria under continuous exposure to insults, and subsequently elicits irreversible damage for both mtDNA and nDNA, and mutation of proto-oncogenes and tumor-suppressor genes, promoting cell transformation and onset of carcinogenesis.^131^ Additionally, mtROS could also promote the survival, angiogenesis, and proliferation of tumor cells by activating NF-κB and stabilize HIF-1α.^132,133^ ROS-induced cell death could further amplify hepatic inflammation by the release of DMAPs and formation of a ROS-related vicious inflammatory loop, resulting in an oxidative and inflammatory microenvironment.^34^ This microenvironment has been proven to favor the development of HCC or CCA and is essential to direct lineage commitment in liver cancer.^134^ A recent study showed that the level of Kupffer cell-derived ROS is particularly higher in human CCA and surrounding hepatocytes, which causes JNK-dependent cholangiocellular proliferation and oncogenic transformation.^135^ However, the involvement of ROS in liver cancer cannot be interpreted by a simple notion of “pro-carcinogenesis.” For instance, due to depletion of mitochondrial GSH, the hypoxic environment within the tumor can be reversed from pro-carcinogenesis to anti-carcinogenesis by overproduction of mtROS, whereby tumor cells are preferentially killed.^136^

## THERAPEUTIC STRATEGIES

### Silymarin/Silibinin

Silymarin is a natural extract from milk thistle (*Silybum marianum*) that has been used for centuries for treating liver diseases. Silymarin is a complex mixture of compounds in which silibinin represents the most prevalent and biologically active structural component.^137^ Silymarin is a potent ROS/RNS scavenger and exhibits hepatoprotective properties in various experimental models.^138,139^ Production of O_2_^−^ and NO in isolated rat Kupffer cells could be effectively inhibited by silibinin.^139^ Silymarin can also enhance the enzymatic antioxidant defense system by increasing GSH synthesis and attenuate acetaminophen hepatoxicity in mice.^138^ A recent study identified silymarin as an inducer of Nrf2 and regulator of the redox signaling pathway.^140^ Additionally, by blocking the activation of NF-κB and inflammatory metabolites, silymarin is capable of restraining the generation of cytokines and thus reducing liver fibrosis induced by carbon tetrachloride toxicity.^141^ Numerous clinical investigations have been conducted to evaluate the hepatoprotective effect of silymarin in liver diseases such as NAFLD, NASH, ALD, drug-induced liver injury, and viral hepatitis. For instance, in a randomized and double-blind study in patients with chronic ALD, a 6-mo treatment of silymarin significantly restored the diminished expression of SOD, increased the level of serum GSH peroxidase, and reduced the serum malondialdehyde.^142^ In contrast, another 2-y period and multicenter trial indicated that silymarin has no beneficial effect on either liver function or mortality in ALD patients with cirrhosis.^143^ This paradox may arise from the various administration periods and dosage of silymarin in different studies. Besides, the distinct silymarin formulations used should also be considered as the oral bioavailability of crude silymarin extract is far lower than Eurosil 85, a commercial formulation of silymarin, which has been applied widely in recent trials. Altogether, silymarin represents a promising therapy for chronic liver diseases which remains to be confirmed in more clinical trials.

### Vitamin E

Vitamin E is a lipid-soluble antioxidant, which has been extensively investigated during the last decade, especially in the field of steatohepatitis. The inherited antioxidative mechanism of vitamin E results from the deposition of ROS/RNS by donating a hydrogen ion from its chromanol ring.^144^ Vitamin E regulates antioxidative enzymes such as manganese SOD and GSH to enhance an antioxidant defense.^145^ In addition to its antioxidative capacity, vitamin E is also able to suppress hepatic fibrogenesis by retarding inflammation and inhibiting apoptosis.^144^ Accumulating evidence demonstrates that vitamin E could reduce lipid accumulation, lipid peroxidation, insulin resistance, oxidative stress, necroinflammation, and thus ameliorate experimental hepatic steatosis.^146,147^ However, the effect of vitamin E in the treatment of steatohepatitis seems controversial. Two large-scale clinical trials, also known as Pioglitazone versus Vitamin E versus Placebo for the Treatment of Non-Diabetic Patients with Nonalcoholic Steatohepatitis and Treatment of Nonalcoholic Fatty Liver Disease in Children, have been conducted. In the Pioglitazone versus Vitamin E versus Placebo for the Treatment of Non-Diabetic Patients with Nonalcoholic Steatohepatitis trial, vitamin E significantly improves the outcome of biopsy-confirmed adult NASH patients without diabetes but did not affect fibrosis.^148^ Hence, vitamin E has been recommended as first-line pharmacotherapy for non-diabetic patients with biopsy-proven NASH.^149^ However, in the Treatment of Nonalcoholic Fatty Liver Disease in Children trial, vitamin E improved both liver histology and NAFLD activity scores in pediatric patients with NASH but failed to reduce the level of transaminases.^150^ Besides, the increased mortality after the treatment of vitamin E in several clinical trials cannot be neglected.^151^

### MitoQ

MitoQ is a ubiquinone-derived mitochondria-specific antioxidant, which attaches to a lipophilic triphenylphosphonium cation and selectively blocks mitochondrial oxidative damage.^152^ MitoQ inhibits lipid peroxidation and hydrogen peroxide-induced apoptosis and protects mitochondria from oxidative damage.^152,153^ Intestinal barrier disruption is a known risk for oxidative stress in liver diseases. As shown in previous studies on intestinal IRI, mitoQ protects the intestinal barrier by meliorating mtDNA damage through the Nrf2/ARE signaling pathway.^154^ It also has a noticeable improvement of mitochondrial function and antiviral CD8 functions in HBV infection.^155^ In mice experiments, mitoQ showed inhibition of HSCs activation and transforming growth factor-beta 1 expression, both key factors in hepatic fibrosis.^156,157^

### Targeting Farnesoid X Receptor

The farnesoid X receptor (FXR) is a nuclear receptor that plays a critical role in energy metabolism, especially in bile acids, fats, and hydrocarbon metabolism. FXR and FXR agonists can reduce oxidative stress, protect mitochondrial function and antagonize the SOD4-activated JNK signaling pathway.^158-160^ Obeticholic acid (OCA), an FXR agonist, is studied in patients with PBC that have an inadequate response to UDCA. Time will show the long-term safety and clinical efficacy of OCA.^161^ OCA was already assessed in NASH patients who had alleviated fibrosis, hepatocellular ballooning, steatosis, and lobular inflammation compared with placebo groups. Still to be analyzed is the effect of OCA on serum cholesterol levels and insulin resistance, as well as the risk of atherogenesis.^162^

### Nonpharmacologic Interventions

Pharmacologic antioxidants have been extensively investigated in experimental models and clinical trials. Beyond that, nonpharmacologic interventions, such as nanocarrier-coated antioxidants, ischemia preconditioning (IPC), gene therapy, machine perfusion, and gaseous supplements, represent unconventional approaches to suppress mitochondrial dysfunction and oxidative stress in liver diseases and transplantation. Notably, the clinical application of current antioxidative therapies is hampered mainly by the instability, poor membrane permeability, short half-life in circulation, and poor solubility and hydrophilicity of the agents.^163^ To solve this, antioxidants can be encapsulated by a nanocarrier to improve its bioavailability. For example, SOD, an enzymatic antioxidant, could be encapsulated into poly(D, L-lactideco-glycolide) nanoparticles and reduced neuron IRI by detoxifying free radicals, of which efficiency of SOD is significantly enhanced.^164^

Antioxidative therapies are of importance to alleviate IRI in liver surgery and could be employed specifically for the treatment of donors, graft livers, and recipients during liver transplantation.^62^ IPC is a surgical procedure that is initiated by a short period of nonlethal ischemia to the target organ before a much longer time of ischemia elicited by surgery such as hepatectomy and graft liver procurement. IPC has been proven to protect the liver from subsequent IRI by regulating redox balance, preventing nuclear damage, and inhibiting cell death.^165^ IPC could increase the tolerance of the fatty rat liver to IRI by activating antioxidative enzymes. Multiple lines of evidence in clinical trials indicate that the IPC on donor could attenuate liver injury following liver transplantation and decrease the mortality of recipients.^166-168^ Machine perfusion remains a promising approach to protect the graft liver from IRI. A recent systematic review demonstrates that both hypothermic machine perfusion and normothermic machine perfusion are superior to static cold storage on improving early graft function in liver transplantation.^169^ Compared with static cold storage, hypothermic machine perfusion could significantly reduce oxidative stress in liver grafts.^170^ Of note, the antioxidative effect of machine perfusion is primarily determined by the temperature because the mitochondrial activity is known to be temperature-dependent.^63^ Indeed, increased production of ROS has been observed in normothermic machine perfusion due to the induction of reverse, instead of forward, electron transfer through the mitochondrial respiratory chain. In contrast, during hypothermic or subnormothermic machine perfusion, the mitochondrial respiratory function is significantly suppressed while the ATP pool is enhanced, leading to less ROS generation.^171^ However, normothermic machine perfusion remains to be superior in terms of its capability to restore metabolism under normothermic condition, thus allowing the monitoring of function of graft liver.^63^ Hence, combination of hypothermic and normothermic machine perfusion represents an optimized protocol to alleviate oxidative injury. A recent study showed that application of normothermic after hypothermic machine perfusion could mitigate oxidative injury and restore mitochondrial function.^172^

## SUMMARY AND CONCLUSION

Endogenous and exogenous hepatic insults are known to cause mitochondrial dysfunction in the liver involving loss of ATP synthesis capacity and increasing ROS/RNS generation. This, in turn, results in oxidative stress and further forms a vicious cycle, which is believed to be the critical pathogenic factors in liver diseases, as well as liver transplantation. Although ROS/RNS are involved in normal cell homeostasis, they are also related to the regulation of apoptosis, necroptosis, and MPT-driven regulated necrosis. Also, ROS/RNS are also capable to indirectly affect hepatocyte function by promoting an inflammatory response and fibrogenesis. The balance of ROS/RNS being either harmful or beneficial largely depends on the intensity and duration of ROS/RNS production. Although numerous antioxidative interventions have been developed for the treatment of liver diseases by either scavenging ROS/RNS or restoring mitochondrial homeostasis, not many showed beneficial effects in clinical trials.^2^ Of note, intake of dietary antioxidant supplements such as beta carotene, vitamin A, and vitamin E might even increase the mortality of patients.^151^ The precise role of mitochondrial dysfunction and oxidative stress in hepatic pathogenesis should be studied in more detail, and the development of therapeutic regimens should be thoroughly examined to not only alleviate detrimental effects of free radicals but also to preserve its biologic function.
